# The miR-132/212 locus: a complex regulator of neuronal plasticity, gene expression and cognition

**Published:** 2016-08-02

**Authors:** Sydney Aten, Katelin F. Hansen, Kari R. Hoyt, Karl Obrietan

**Affiliations:** 1 Department of Neuroscience, Ohio State University, Columbus, OH, 43210, USA; 2 Division of Pharmacology, Ohio State University, Columbus, OH, 43210, USA

**Keywords:** miR-132/212, cognition, memory, learning, hippocampus, neuronal plasticity

## Abstract

The microRNA (miRNA) class of small (typically 22–24 nt) non-coding RNA affects a wide range of physiological processes in the mammalian central nervous system (CNS). By acting as potent regulators of mRNA translation and stability, miRNAs fine-tune the expression of a multitude of genes that play critical roles in complex cognitive processes, including learning and memory. Of note, within the CNS, miRNAs can be expressed in an inducible, and cell-type specific manner. Here, we provide a brief overview of the expression and functional effects of the miR-132/212 gene locus in forebrain circuits of the CNS, and then discuss a recent publication that explored the contributions of miR-132 and miR-212 to cognition and to transcriptome regulation. We also discuss mechanisms by which synaptic activity regulates miR-132/212 expression, how miR-132 and miR-212 affect neuronal plasticity, and how the dysregulation of these two miRNAs could contribute to the development of cognitive impairments.

## The miR-132/212 gene cluster and inducible expression in the CNS

miRNAs have been shown to play a critical role in neuronal development, signaling, and plasticity^[1, 2]^. As with coding genes, miRNA expression within the CNS can be tightly regulated by changes in neuronal activity. Consistent with this, the miR-132/212 locus was initially pulled from a screen designed to identify genes regulated by the CREB/CRE transcriptional pathway^[3]^, a key conduit whereby synaptic activity drives the expression of genes that underlie neuronal plasticity and long-term memory formation^[4, 5]^. Using a combination of ChIP-based profiling and DNase I footprinting assays, Vo *et al*. identified two consensus CRE sites 5′ to the miR-212 transcript and a third site located between miR-212 and miR-132^[3]^.

In mice, the miR-132/212 gene cluster is transcribed from the first intron of a noncoding transcript, AK006051, on Chromosome 11. In addition, an alternate transcript has also been detected, where the miR-132/212 loci are processed from its second exon (both transcript variants have been detected in the brain)^[6]^. In humans, the miR-132/212 gene cluster has similar mature sequences and identical seed sequences to its murine homolog, but it is located within an intergenic region, on Chromosome 17p13.3. miR-132 and miR-212 likely resulted from gene duplication: consistent with this idea, the two miRNAs share identical 8 base seed sequences, although the flanking regions are divergent^[7]^. Given that miRNA base-complementation within the seed region is a key factor in translational inhibition of target mRNAs (reviewed in^[8]^), a good number of the predicted mRNA targets between miR-132 and miR-212 are redundant. However, the miRNA seed sequence flanking regions, the folded structure of the potential mRNA target, and the molecular architecture of the target position within the 3’ UTR also play significant roles in miRNA-mRNA hybridization and in the efficiency of mRNA degradation and translational inhibition^[9, 10]^. Hence, to date, the degree of functional redundancy between these two miRNAs is not known (discussed in detail below).

As noted, neuronal stimulation leads to a marked increase in transcription from the miR-132/212 locus. For example, paradigms such as photic stimulation, associative learning, and seizure induction have all been shown to increase miR-132/212 expression in the CNS^[11–15]^, and, consistent with the presence of the CRE motifs, neuronal induction has been shown to be mediated in part by the CREB/CRE transcriptional pathway^[3, 7]^. Interestingly, an increase in miR-132 within dendrites has been detected following neuronal stimulation and as a function of neuronal development^[16, 17]^.

## Processing, stability and functional expression of miR-132 and miR-212

Several studies have focused on the processing and stability of the pri-miR-132/212 transcript and the maturation of miR-132/212 in neurons. Notably, Remenyi *et al*. used a combination of high throughput RNA sequencing and quantitative PCR profiling to show that BDNF stimulates lasting upregulation of both miR-132-3p and miR-212-3p (and their corresponding antisense 5p strands)^[7]^. However, Magill *et al*. used ratiometric miRNA fluorescent reporters in immature hippocampal neurons, and found that, of the four possible miRNAs processed from the miR-132/212 locus, only miR-132-3p exhibited functional expression^[18]^. Further, Remenyi *et al*. recently reported that miR-132 is markedly overexpressed relative to miR-212 in a wide variety of murine tissue types^[19]^. Interestingly, this difference in miR-132 expression appears to be a consequence of the uneven processing of the miRNAs, a result ascribed to the miR-132 loop structure and its functional interaction with numerous RNA binding proteins, which likely leads to more efficient pri-miR-132 processing^[19]^. However, even with this difference in the expression of the two miRNAs, it is still important to note that a number of studies have shown inducible miR-212 expression and functionality within the mature nervous system^[20, 21]^. Clearly, additional studies will be required to examine how miRNA expression from the miR-132/212 locus is regulated as a function of both neuronal cell-type and development.

## The role of miR-132 in synaptic plasticity and neuronal morphology

Vo *et al*. identified miR-132 (and characterized its CREB-regulated expression) and reported that miR-132 regulates the outgrowth of neuronal processes^[3]^. This finding provided a framework for morpho-metric-based studies that placed miR-132 into ever more complex functional contexts (for extensive discussions of miR-132/212 in the nervous system readers are referred to several excellent review articles^[22–24]^). Along these lines, abrogation of miR-132 expression (via conditional deletion of the miR-132/212 locus or retroviral knockdown of miR-132) led to a decrease in dendritic arborization of adult-born developing neurons of the subgranular zone of the dentate gyrus^[18]^. Likewise, knockdown approaches in developing brain slices revealed that miR-132 modulates dendritic arborization^[16]^. Tognini *et al*. reported that miR-132 is induced by input to the visual cortex and that it regulates developmentally-gated ocular dominance plasticity and neuronal spine density^[25]^. Finally, in the mature nervous system, transgenic overexpression of miR-132 was found to increase spine density in CA1 dendrites^[26]^. These effects on neuronal morphology have been associated with the expression of a number of miR-132 targets such as p250GAP, MeCP2, and MMP-9^[3, 16, 26, 27]^. Consistent with these studies, miR-132 has been shown to affect both basal and evoked synaptic transmission^[17, 28–30]^. Given the key role that activity-dependent morphological and functional plasticity plays in adaptive processes such as learning and memory, these findings raised the prospect that miR-132/212 could form a conduit by which changes in synaptic activity modulate complex behavioral states.

## miR-132/212 and the modulation of learning and memory

Having established that the miR-132/212 locus is regulated by neuronal activity, and that miR-132 regulates key functional features of neurons (e.g., dendrite morphology and dendrite spine density), a logical next step was to test the role of miR-132/212 in learning and memory. To this end, Hansen *et al*. employed a transgenic mouse model to show that an ~ 5-fold *overexpression* of miR-132 in excitatory forebrain neurons leads to significant deficits in recognition and spatial memory^[26]^. Consistent with this, lentiviral-based *overexpression* of miR-132 in the perirhinal cortex was found to reduce recognition memory capacity in the novel object recognition (NOR) task^[31]^. As with transgenic miR-132 overexpression animals, germline miR-132/212 knockout mice exhibited deficits in recognition and spatial memory^[32]^. Although a similar effect with both gain-of-function and loss-of-function approaches is, on the surface, counter-intuitive, it did raise the possibility that the expression from the miR-132/212 locus must be maintained within a tightly regulated range to ensure normal functionality. Indeed, when Hansen *et al*. returned to the question of miR-132 and cognition, they utilized a Tet-off system to titer transgenic miR-132 to levels that paralleled the levels observed following a learning paradigm (i.e. ~ 2-fold above basal) and found that cognitive capacity was enhanced^[13]^. Consistent with these findings, maintaining miR-132 within a limited/physiological range is also essential for visual cortex plasticity. Tognini *et al*. showed that infusing a miR-132 mimic oligonucleotide into the visual cortex blocked ocular dominance plasticity and increased the number of mushroom spines^[25]^, while Mellios *et al*. showed that inhibition of miR-132 not only prevented ocular dominance plasticity after monocular deprivation, but also led to an increase in immature spines^[11]^. Collectively, these studies established a functional role for miR-132 in cognition and plasticity. However, key questions regarding the role of miR-212 in cognition had not been examined. Further, the relative contributions of miR-132 and miR-212 to gene expression in the CNS had not been tested.

## Hansen et al. (2016): miR-132/212, learning and memory, and the hippocampal transcriptome

At this point, we turn to the recent paper by Hansen *et al*. that brought together a combination of novel mouse models and RNA-seq profiling methods to assess the distinct contributions of miR-132 and miR-212 to gene expression and to hippocampal function^[33]^. Again, given that both miRNAs have a common seed sequence and are expressed from the same noncoding transcript, it has been difficult to assess the unique contribution of each miRNA to the physiology of the forebrain.

For this study three separate mouse lines were used: a floxed miR-132/212 deletion line where Cre recombinase was driven under the control of the CaMKII promoter (*CaMKII-Cre::miR-132/-212^f/f^*), and two transgenic Tet-responsive mouse lines that drive the expression of either miR-132 or miR-212 via a CaMKII-tTA line^[34]^ (*CaMKII-tTA::miR-132; CaMKII-tTA::miR-212*, respectively). These approaches allowed for the selective deletion of the miR-132/212 locus and the overexpression of each miRNA in the same populations of excitatory forebrain neurons. Consistent with this, expression analysis of the *CaMKII-Cre::miR-132/-212^f/f^* line and the transgenic lines confirmed that targeting was restricted to the major excitatory cell populations of the hippocampus (e.g., CA3, CA1, and GCL)^[26, 33]^.

Focusing first on the cognition tests, Hansen *et al*. demonstrated that the deletion of the miR-132/212 locus resulted in significant impairments in the novel object recognition task, in the Barnes maze paradigm, and in contextual and tone fear conditioning—suggestive of deficits in recall memory and spatial memory, respectively^[33]^. These data complemented prior work by Hansen *et al*. showing that the transgenic overexpression of miR-132 (to levels that approximate those observed following a learning paradigm) enhanced cognitive capacity^[13]^, and recent work by Hernandez-Rapp *et al*. who reported cognitive deficits in a miR-132/212 knockout mouse line^[32]^.

Turning to miR-212, to date, no study had tested its in cognition. Using the novel object recognition task, Hansen *et al*. found that miR-212 transgenic mice showed a significantly *reduced* capacity to discriminate between novel and familiar objects, suggesting that miR-212 has the potential to regulate learning and memory efficacy^[33]^. More recently, our lab has found that the miR-212 transgenic mouse line has a subtle anxiety phenotype (assessed using the elevated plus maze and open field test), and deficits in spatial memory (assessed using the Barnes maze) (unpublished observations). Importantly, in all of these studies, expression of the miR-212 transgene was not titered with doxycycline. These results can be viewed in multiple ways. In the most straightforward assessment, these findings indicate that miR-212 functions as a negative regulator of recall memory, and thus, would run counter to the findings for the targeted deletion of the miR-132/212 locus. However, this interpretation should be viewed with a bit of caution, given prior work showing that, in the absence of doxycycline titered transgene expression, miR-132 transgenic mice also exhibited recall memory deficits^[26]^. Again, improved cognitive ability was only found after transgenic miR-132 was titered (with doxycycline) to levels that match the induction level following a spatial learning paradigm^[13]^. Thus, a similar doxycycline treatment approach may be needed to effectively model the function of endogenous miR-212. Clearly, further work with the miR-212 transgenic mouse line will be required to fully characterize its potential roles in learning and memory.

Although a number of mRNA targets, including *Mmp-9, p250GAP*, and *Mecp2*, which influence neuronal morphology^[27, 3, 16, 28, 18, 26]^, have been described over the past several years, there are large gaps in our understanding of the transcriptome-wide effects (both direct targets and indirect network effects) of miR-132 and miR-212. Likewise, data regarding the degree of target redundancy between miR-132 and miR-212 has not been systematically examined. Hansen *et al*. attempted to examine these questions by harnessing the power of RNA-seq profiling to investigate the transcriptional profiles of the three noted mouse lines: *CaMKII-Cre::miR-132/-212^f/f^; CaMKII-tTA::miR-132; CaMKII-tTA::miR-212*^[33]^. These datasets were then used to generate an intersectional analysis of miR-132 and miR-212 targeted genes. The prediction from these studies was that mRNA targets would be upregulated in miR-132/212 knockout mice and downregulated in the transgenic overexpression animals. Logically, if the two miRNAs do exhibit target redundancy, then the same mRNAs should be downregulated in both transgenic lines; and conversely, non-overlapping mRNA populations would suggest functional divergence between the miRNAs.

Hippocampal RNA-seq profiling revealed that the miR-132/212 knockout yielded 1,138 significantly upregulated transcripts and 886 significantly downregulated transcripts. In the knockout mouse line, upregulated transcripts were viewed as potential direct targets whereas the downregulated transcripts were most likely the result of indirect gene network effects of the miRNAs. Transgenic overexpression of miR-132 revealed 1,266 significantly upregulated and 928 downregulated transcripts, whereas miR-212 overexpression generated 78 upregulated and only 58 downregulated genes^[33]^. Ontological analysis identified a number of functional grouping associations with synaptic transmission and neuronal morphogenesis—both of which are thought to be key processes that underlie learning and memory. Intersectional analysis of the upregulated dataset from the miR-132/212 knockout line with the miR-132 predicted targets (based on datasets curated from microRNA.org), and the downregulated RNA-seq datasets generated from the miR-132 transgenic mouse line identified 18 genes that met the 3-way intersection criteria. Interestingly, a similar analysis for miR-212 identified only one gene (*Stx1a*); downregulation of STX1A was confirmed in the miR-212 transgenic mouse line using immunohistochemical labeling^[33]^.

One of the most surprising findings of the RNA-seq studies was the limited number of transcripts that were downregulated in the miR-212 transgenic mice (58), and of these, only one transcript met the 3-way intersectional criteria (of note, 13 transcripts met a 2-way intersectional criteria when comparing between the miR-212 transgenic mice and the microRNA.org predicted targets). There are several possible reasons why so few targets met the 3-way criteria. One potential factor/cause is that the mRNA profiling was performed in mice with tonically high levels of miR-212; this high ‘baseline’ level of the transgene may have resulted in compensatory upregulation of mRNA targets. With this in mind, a nice addition to the study would have been to examine the mRNA profile following a transient increase in miR-212 (this suggestion can also be applied to the examination of transgenic miR-132). Another possibility is that miR-212 simply has limited functionality within the hippocampus (i.e., limited miR-212 expression would result in an absence of direct targets).

With respect to the miR-132 dataset, it is worth noting that some previously validated miR-132 targets such as *p250GAP, Mmp-9, Paip2A, Sirt1*, and *Ptbp2* were not found to be significantly downregulated in the RNA-seq screens. One potential reason for this could have come from high variability across biological samples and/or limited enrichment that kept the fold expression under the study cut-off. Certainly, other factors, including time-of-day (i.e., circadian) effects, age of the animals, and the exclusive focus on the hippocampus could have also impacted the mRNA targeting datasets. Even with these caveats in mind, this study provides a unique picture of the functional complexity of the miR-132/212 locus within the hippocampus.

## Future Directions

Substantial progress has been made in elucidating the expression patterns and functional roles of miR-132 and miR-212. By virtue of their inducible expression, their ability to affect neuronal morphology and their potential to shape learning and memory capacity, these miRNAs are poised to play key roles in modulating the core functional features of the CNS. Despite this growing body of knowledge, many questions remain regarding the key characteristics and mRNA targeting properties of miR-132 and miR-212. Further, miR-132/212 dysregulation has been associated with a number of neurodegenerative disorders, including Alzheimer’s disease^[35–37]^ and Huntington’s disease^[38, 39]^, and neurocognitive disorders, including autism^[40]^, Rett syndrome^[41]^, and schizophrenia^[42–44]^. Hence, a deeper understanding of regulation and function of miR-132 and miR-212 could provide novel therapeutic approaches to treat an array of neurological disorders.

## Abbreviations

CNS: central nervous system
CRE: cAMP-response element
CREB: cAMP-responsive element binding protein
ChIP: chromatin immunoprecipitation
UTR: untranslated region
CaMKII: Ca2+/calmodulin-dependent protein kinase II
BDNF: brain-derived neurotrophic factor
CA1: cornu ammonis field 1 of the hippocampus
CA3: cornu ammonis field 3 of the hippocampus
GCL: granule cell layer of the hippocampus

## References

[1] Im HI, Kenny PJ (2012). MicroRNAs in neuronal function and dysfunction. Trends Neurosci.

[2] Fiore R, Khudayberdiev S, Saba R, Schratt G (2011). MicroRNA function in the nervous system. Prog Mol Biol Transl Sci.

[3] Vo N, Klein ME, Varlamova O, Keller DM, Yamamoto T, Goodman RH (2005). A cAMP-response element binding protein-induced microRNA regulates neuronal morphogenesis. Proc Natl Acad Sci U S A.

[4] Sakamoto K, Karelina K, Obrietan K (2011). CREB: a multifaceted regulator of neuronal plasticity and protection. J Neurochem.

[5] Barco A, Marie H (2011). Genetic approaches to investigate the role of CREB in neuronal plasticity and memory. Mol Neurobiol.

[6] Ucar A, Vafaizadeh V, Jarry H, Fiedler J, Klemmt PA, Thum T (2010). miR-212 and miR-132 are required for epithelial stromal interactions necessary for mouse mammary gland development. Nat Genet.

[7] Remenyi J, Hunter CJ, Cole C, Ando H, Impey S, Monk CE (2010). Regulation of the miR-212/132 locus by MSK1 and CREB in response to neurotrophins. Biochem J.

[8] Valencia-Sanchez MA, Liu J, Hannon GJ, Parker R (2006). Control of translation and mRNA degradation by miRNAs and siRNAs. Genes Dev.

[9] Cloonan N (2015). Re-thinking miRNA-mRNA interactions: Intertwining issues confound target discovery. BioEssays.

[10] Didiano D, Hobert O (2006). Perfect seed pairing is not a generally reliable predictor for miRNA-target interactions. Nat Struct Mol Biol.

[11] Mellios N, Sugihara H, Castro J, Banerjee A, Le C, Kumar A (2011). miR-132, an experience-dependent microRNA, is essential for visual cortex plasticity. Nat Neurosci.

[12] Cheng HY, Papp JW, Varlamova O, Dziema H, Russell B, Curfman JP (2007). microRNA modulation of circadian-clock period and entrainment. Neuron.

[13] Hansen KF, Karelina K, Sakamoto K, Wayman GA, Impey S, Obrietan K (2013). miRNA-132: a dynamic regulator of cognitive capacity. Brain Struct Funct.

[14] Nudelman AS, DiRocco DP, Lambert TJ, Garelick MG, Le J, Nathanson NM (2010). Neuronal activity rapidly induces transcription of the CREB-regulated microRNA-132, in vivo. Hippocampus.

[15] Jimenez-Mateos EM, Bray I, Sanz-Rodriguez A, Engel T, McKiernan RC, Mouri G (2011). miRNA Expression profile after status epilepticus and hippocampal neuroprotection by targeting miR-132. Am J Pathol.

[16] Wayman GA, Davare M, Ando H, Fortin D, Varlamova O, Cheng HY (2008). An activity-regulated microRNA controls dendritic plasticity by down-regulating p250GAP. Proc Natl Acad Sci U S A.

[17] Pathania M, Torres-Reveron J, Yan L, Kimura T, Lin TV, Gordon V (2012). miR-132 enhances dendritic morphogenesis, spine density, synaptic integration, and survival of newborn olfactory bulb neurons. PLoS One.

[18] Magill ST, Cambronne XA, Luikart BW, Lioy DT, Leighton BH, Westbrook GL (2010). microRNA-132 regulates dendritic growth and arborization of newborn neurons in the adult hippocampus. Proc Natl Acad Sci U S A.

[19] Remenyi J, Bajan S, Fuller-Pace FV, Arthur JS, Hutvagner G (2016). The loop structure and the RNA helicase p72/DDX17 influence the processing efficiency of the mice miR-132. Sci Rep.

[20] Im HI, Hollander JA, Bali P, Kenny PJ (2010). MeCP2 controls BDNF expression and cocaine intake through homeostatic interactions with microRNA-212. Nat Neurosci.

[21] Hollander JA, Im HI, Amelio AL, Kocerha J, Bali P, Lu Q (2010). Striatal microRNA controls cocaine intake through CREB signalling. Nature.

[22] Wanet A, Tacheny A, Arnould T, Renard P (2012). miR-212/132 expression and functions: within and beyond the neuronal compartment. Nucleic Acids Res.

[23] Tognini P, Pizzorusso T (2012). MicroRNA212/132 family: molecular transducer of neuronal function and plasticity. Int J Biochem Cell Biol.

[24] Bicker S, Lackinger M, Weiß K, Schratt G (2014). MicroRNA-132-134, and-138: a microRNA troika rules in neuronal dendrites. Cell Mol Life Sci.

[25] Tognini P, Putignano E, Coatti A, Pizzorusso T (2011). Experience-dependent expression of miR-132 regulates ocular dominance plasticity. Nat Neurosci.

[26] Hansen KF, Sakamoto K, Wayman GA, Impey S, Obrietan K (2010). Transgenic miR132 alters neuronal spine density and impairs novel object recognition memory. PLoS One.

[27] Jasińska M, Miłek J, Cymerman IA, Łęski S, Kaczmarek L, Dziembowska M (2015). miR-132 regulates dendritic spine structure by direct targeting of matrix metalloproteinase 9 mRNA. Mol Neurobiol.

[28] Impey S, Davare M, Lesiak A, Fortin D, Ando H, Varlamova O (2010). An activity-induced microRNA controls dendritic spine formation by regulating Rac1-PAK signaling. Mol Cell Neurosci.

[29] Lambert TJ, Storm DR, Sullivan JM (2010). MicroRNA132 modulates short-term synaptic plasticity but not basal release probability in hippocampal neurons. PLoS One.

[30] Luikart BW, Bensen AL, Washburn EK, Perederiy JV, Su KG, Li Y (2011). miR-132 mediates the integration of newborn neurons into the adult dentate gyrus. PLoS One.

[31] Scott HL, Tamagnini F, Narduzzo KE, Howarth JL, Lee YB, Wong LF (2012). MicroRNA-132 regulates recognition memory and synaptic plasticity in the perirhinal cortex. Eur J Neurosci.

[32] Hernandez-Rapp J, Smith PY, Filali M, Goupil C, Planel E, Magill ST (2015). Memory formation and retention are affected in adult miR-132/212 knockout mice. Behav Brain Res.

[33] Hansen KF, Sakamoto K, Aten S, Snider KH, Loeser J, Hesse AM (2016). Targeted deletion of miR-132/-212 impairs memory and alters the hippocampal transcriptome. Learn Mem.

[34] Mayford M, Bach ME, Huang YY, Wang L, Hawkins RD, Kandel ER (1996). Control of memory formation through regulated expression of a CaMKII transgene. Science.

[35] Hebert S, Sergeant N, Buee L (2012). MicroRNAs and the Regulation of Tau Metabolism. Int J Alzheimers Dis.

[36] Lau P, Bossers K, Janky R, Salta E, Frigerio CS, Barbash S (2013). Alteration of the microRNA network during the progression of Alzheimer’s disease. EMBO Mol Med.

[37] Wong HK, Veremeyko T, Patel N, Lemere CA, Walsh DM, Esau C (2013). De-repression of FOXO3a death axis by microRNA-132 and-212 causes neuronal apoptosis in Alzheimer’s disease. Hum Mol Genet.

[38] Johnson R, Buckley NJ (2009). Gene dysregulation in Huntington’s disease: REST, microRNAs and beyond. Neuromolecular Med.

[39] Lee ST, Chu K, Im WS, Yoon HJ, Im JY, Park JE (2011). Altered microRNA regulation in Huntington’s disease models. Exp Neurol.

[40] Abu-Elneel K, Liu T, Gazzaniga FS, Nishimura Y, Wall DP, Geschwind DH (2008). Heterogeneous dysregulation of microRNAs across the autism spectrum. Neurogenetics.

[41] Klein ME, Lioy DT, Ma L, Impey S, Mandel G, Goodman RH (2007). Homeostatic regulation of MeCP2 expression by a CREB-induced microRNA. Nat Neurosci.

[42] Kim AH, Reimers M, Maher B, Williamson V, McMichael O, McClay JL (2010). MicroRNA expression profiling in the prefrontal cortex of individuals affected with schizophrenia and bipolar disorders. Schizophr Res.

[43] Miller BH, Zeier Z, Xi L, Lanz TA, Deng S, Strathmann J (2012). MicroRNA-132 dysregulation in schizophrenia has implications for both neurodevelopment and adult brain function. Proc Natl Acad Sci U S A.

[44] Perkins DO, Jeffries CD, Jarskog LF, Thomson JM, Woods K, Newman MA (2007). microRNA expression in the prefrontal cortex of individuals with schizophrenia and schizoaffective disorder. Genome Biol.

